# Dietary Frankincense (*Boswellia serrata*) Oil Modulates the Growth, Intestinal Morphology, the Fatty Acid Composition of Breast Muscle, Immune Status, and Immunoexpression of CD3 and CD20 in Broiler Chickens

**DOI:** 10.3390/ani13060971

**Published:** 2023-03-07

**Authors:** Shimaa A. Amer, Ahmed Gouda, Gehan K. Saleh, Arwa H. Nassar, Abdel-Wahab A. Abdel-Warith, Elsayed M. Younis, Dalia E. Altohamy, Maha S. Kilany, Simon J. Davies, Anaam E. Omar

**Affiliations:** 1 Department of Nutrition and Clinical Nutrition, Veterinary Medicine Faculty, Zagazig University, Zagazig 44511, Egypt; 2 Department of Animal Production, Agricultural & Biological Research Division, Center of National Research, Dokki, Cairo 11865, Egypt; 3 Biochemistry Department, Animal Health Research Institute (AHRI) (Mansoura Branch) Agriculture Research Center (ARC), Dokki, P.O. Box 246, Giza 12618, Egypt; 4 Food Hygiene Department, Animal Health Research Institute (AHRI) (Mansoura Branch) Agriculture Research Center (ARC), Dokki, P.O. Box 246, Giza 12618, Egypt; 5 Department of Zoology, College of Science, King Saud University, P.O. Box 2455, Riyadh 11451, Saudi Arabia; 6 Department of Pharmacology, Central Laboratory, Faculty of Veterinary Medicine, Zagazig University, Zagazig 44511, Egypt; 7 Department of Histology and Cytology, Veterinary Medicine Faculty, Zagazig University, Zagazig 44511, Egypt; 8 School of Science and Engineering, National University of Ireland Galway Republic of Ireland, H91 TK33 Galway, Ireland

**Keywords:** broiler chickens, growth, frankincense oil, histomorphology, biochemical indices, immune, fatty acid profile

## Abstract

**Simple Summary:**

The expanding knowledge of risks posed by antibiotic resistance in the past decades has led the livestock industry to encourage antibiotic-free production. The search for alternatives to antibiotic growth stimulants has shown a rapid increase. The current work assessed the outcomes of dietary frankincense resin (*Boswellia serrata*) oil inclusion (0, 200, 400, or 600 mg kg^−1^ diet) on the performance, carcass traits, the fatty acid content of breast muscle, protein profile, thyroid hormones, and immune status of broiler chickens. The collective outcomes of this experiment suggested that frankincense oil supplementation exerted a positive effect on the growth and intestinal histology of broilers, and enriched the n-3 and n-6 fatty acid content and enhanced their immunity.

**Abstract:**

This investigation explored the impact of dietary frankincense resin oil (FO) on growth performance parameters, intestinal histomorphology, fatty acid composition of the breast muscle, and the immune status of broilers. We allotted 400, three-day-old, male chicks (Ross 308 broiler) into four treatment groups (ten replicates/group; ten chicks/replicate). They were fed a basal diet with different concentrations of FO (0, 200, 400, and 600 mg kg^−1^). FO supplementation increased the overall body weight (BW) and body weight gain (BWG) by different amounts, linearly improving the feed conversion ratio with the in-supplementation level. Total feed intake (TFI) was not affected. Growth hormones and total serum protein levels also linearly increased with the FO level, while albumin was elevated in the FO600 group. Moreover, total globulins increased linearly in FO400 and FO600 treatment groups. Thyroxin hormone (T3 and T4) levels increased in all FO treatment groups without affecting glucose and leptin serum values. Different concentrations of FO supplementation in the diet increased the activities of Complement 3, lysozyme, and interleukin 10 levels in the serum. Dietary FO in broilers increased the total percentage of n-3 and n-6 fatty acids. It also increased the ratio of n-3 to n-6 linearly and quadratically. Additionally, FO supplementation led to the upregulation of immune clusters of differentiation 3 and 20 (CD3 and CD20) in the spleen, along with improving most of the morphometric measures of the small intestine. In conclusion, FO up to 600 mg kg^−1^ as a feed additive in broiler chicken production is valuable for promoting their growth, intestinal histomorphology, and immune status along with enriching breast muscle with polyunsaturated fatty acids (PUFA).

## 1. Introduction

For an extended period, essential oils from aromatic and medicinal plants have been widely employed in poultry production due to their helpful pharmacologically active components and their minimum side effects, as mentioned in the WHO’s recommendations [1,2]. One such compound is Frankincense, which contains numerous biologically active constituents [3]. Frankincense is obtained from the *Boswellia* genus that belongs to the *Burseraceae* family. It is an aromatic resin that solidifies to form a yellow–brown granular substance, identified as olibanum [4]. Commercially, it is traded as granules, pellets, or powder [5]. Numerous species of *Boswellia* have been identified, including *Boswellia sacra*, *B. frereana*, *B. Serrata* (*B. thurifera*, Indian frankincense), and *B. papyrifera*. These species produce frankincense oil with different compositions, which largely depend on the geographical source, conditions of harvesting, and climate [6]. Enrichment of poultry diets with frankincense (*Boswellia serrata*) as a natural product exerted many beneficial effects on productive performance, including increased body weight, the efficiency of feed consumption and absorption, enhanced meat quality, i.e., meat production with low-fat content and no residue, along with increased serum levels of globulin, lymphocyte numbers, and immunity [7].

The principal constituent of frankincense resin is its oil content (60%), which includes monoterpenes (13.1%), sesquiterpenes (1%), and diterpenes (42.5%). FO encourages pancreatic enzyme secretion, increases protein and energy digestibility, and reduces nitrogen, ammonia, and microbial metabolite losses [8]. A decrease in the uric acid level in broilers’ blood by FO suggests high efficiency in protein absorption and lower loss of endogenous protein [7]. FO also exhibits immunostimulatory and antibacterial activity against many gram-positive and gram-negative bacteria [9,10]. Moreover, broilers’ diet supplementation with Frankincense positively influences the microbes (microbiome) in the gastrointestinal tract, increasing the counts of beneficial bacteria *Lactobacillus* and *Enterococcus* but decreasing the pH of the digestive tract. During subclinical infection, this reduces microorganisms’ energy and protein consumption in the host, therefore, enhancing growth and minimizing the flux of ammonia and immune intercessor [11,12]. In vitro, *Boswellia serrata* oil exhibits antimicrobial activities with maximum inhibition zone [13,14,15], antibacterial and antifungal activities [16,17], anti-inflammatory and anti-diarrheal activities associated with anti-cholinergic effects [18], hepatoprotective and antioxidant activities [5,15], and neuroprotective activity [19,20]. It is also helpful for asthma patients as it eases breathing and has a calming effect during cough, cold, and inflammation of bronchus and larynges [21]. These beneficial effects are associated with several antioxidant compounds present in FO, including mono and diterpenes, ethyl acetate, octyl acetate, and methyl anisole [11].

However, there is a lack of studies exploring FO supplementation on broiler performance. Thus, our study determined the effect of FO dietary supplementation on selected performance parameters of broiler diets, such as intestinal histomorphology, the immune status of birds, the fatty acid profile of breast muscle, blood biochemical parameters, and immunoexpression of CD3 and CD20 in the spleen. The study replicated a typical broiler production system within an experimental facility, thus providing a practical basis for interpretation and potential applications.

## 2. Material and Methods

### 2.1. Gas Chromatography–Mass Spectrometry (GC-MS) Analysis of FO

Frankincense resin (*Boswellia serrata*) oil was obtained from Organic Egypt Company (Cairo, Egypt). The active compounds of FO were determined using a Trace GC1310-ISQ Mass Spectrometer (Thermo Scientific, Austin, TX, USA), with a direct capillary column TG–5MS (30 m × 0.25 mm × 0.25 µm film thickness), following the previous description of Amer, et al. [22].

### 2.2. Birds

The experiments were performed at the Faculty of Veterinary Medicine in the Poultry Research unit, Zagazig University, Egypt. The Ethical approval for the experimental protocol was obtained from the Institutional Animal Care and Use Committee of Zagazig University, Egypt (Approval No. ZU-IACUC/2/F/152/2022).

We obtained 400, one-day-old, Ross 308 broiler chicks from a local hatchery. Chicks were reared in an open, well-ventilated house with sawdust bedding (7 birds/m^2^). During the first week, the building temperature was set at 34 °C, which was reduced gradually to 25 °C at the end of the experiment. Initially, the illumination regime was set to 23 h light/1 h dark condition and then changed to 20 h light/4 h dark condition until the end of the experiment. Standard health and vaccination programs were implemented against Newcastle and Gumboro diseases. The chicks were monitored daily for any health problems.

### 2.3. Experimental Design and Diets

On arrival, chicks were exposed to a three-day adaptation period, wherein they were fed a control diet to attain an average initial weight of 99.18 ± 0.14 g. Next, the birds were randomly assigned into four treatment groups (100 chicks each; ten replicates/group; ten chicks/replicate). The experimental groups were as follows: T1 (control group), a basal diet without FO addition (FO0); T2, basal diet + 200 mg FO/kg (FO200); T3, basal diet + 400 mg FO/kg (FO400); T4, basal diet + 600 mg FO/kg (FO600). FO was mechanically mixed with the feed ingredients and offered to the birds in a mashed form. The experiment lasted for 35 days. The feeding period was divided into the following three periods: starter (4th–10th day), grower (11th–23rd day), and finisher periods (24th–35^th^ day). Throughout the experiment, feed and water were added ad libitum. We formulated the ration for each feeding period (starter, grower, and finisher) according to Ross’s manual guide [23], which is illustrated in Table 1.

### 2.4. Growth Performance

To calculate the average initial BW, birds were weighed individually on their 4th day, and then to determine their BW and **BWG**; they were reweighed at 10, 23, and 35 days. The feed intake (**FI**) was calculated as the difference between the amount of feed offered and the amount of feed residue left at the end of each feeding period, which was divided by the number of birds in each replicate. The feed conversion ratio (**FCR**) was computed as per the following equation:

$$FCR=\frac{FI}{BWG}$$

### 2.5. Percentage Calculations of the Dressing, Internal Organs, and Immune Organs

To calculate the percentage of dressing, nine chicks were chosen from each group, weighed, and euthanized using cervical dislocation [24]. The carcasses were plucked, eviscerated, and weighed to determine the carcass weight. The percentage of dressing was determined as follows:

Dressing % = Carcass weight (g)/Live BW (g) × 100.

While the percentages of the internal organs (liver, gizzard, and intestine) and immune organs (spleen and bursa of Fabricius) were determined as follows:

Weights of the organs (g)/the live weight (g) × 100.

### 2.6. Sampling

For further analyses, three birds were randomly chosen from each replicate (n = 30/group) and euthanized using cervical dislocation [24]. Blood samples (n = 30/group) were collected into sanitized tubes without anticoagulant and were allowed to clot at room temperature. The tubes were then centrifuged at 3500 rpm for 15 min to separate the serum. The separated serum samples were stored at –20 °C until further chemical analysis. Samples from breast muscles (n = 5/group), intestinal samples from the duodenum, jejunum, and ileum (n = 10/group, 2 cm), and spleen samples (n = 10/group) were collected for fatty acid analysis, histomorphological examination, and immunohistochemistry, respectively.

### 2.7. Fatty Acid Analysis of the Breast Muscle

For the fatty acid analysis, five breast muscle samples (50 g/sample) were collected from each group. A chloroform/methanol (2:1, *v*/*v*) solvent method, as described by Belitz et al., was used to extract fat [2].The extracted fatty acids were then measured according to AOAC [25].

### 2.8. Intestinal Histology and Morphometric Measures

Intestinal specimens were stored in 10% neutral buffered formaldehyde for 72 h and then dehydrated using an ascending grade of ethanol (75–100%). Next, they were treated with xylol I and II and later embedded in paraffin. Finally, the samples were sliced into 4 µm longitudinal and cross-sections using a microtome (Leica RM 2155, England). Slides were stained using Hematoxylin and Eosin (H&E) [26]. The morphometric dimensions were measured as per the description of Amer, et al. [27].

### 2.9. Blood Biochemical Parameters

The total serum protein level was determined according to the procedures of Grant [28]. The albumin level was assessed according to Doumas, et al. [29]. The serum globulin level was computed by subtracting albumin from total protein values as per Doumas, et al. [30]. The serum glucose value was evaluated using an automatic biochemical analyzer (Robotnik Prietest ECO, India) [31]. The hormones of the thyroid gland (triiodothyronine (T3) and thyroxin (T4)), leptin, and growth hormones (GH) were assessed using chicken ELISA kits (My BioSource Co., San Diego, CA, USA, with Cat. No. MBS269454, MBS265796, MBS025331, and MBS266317, respectively) following the manufacturer’s instructions.

### 2.10. Immunological Parameters

Interleukin 10 (IL10) was quantified using a specific ELISA assay kit (MyBioSource, San Diego, CA, USA) (Cat. No. MBS701683). C3 level was determined using another ELISA kit (Life Span Biosciences, Inc., Seattle, WA, USA) (Cat. No. LS-F9287). Lysozyme activity was measured as per Lie, et al. [32].

### 2.11. Immunohistochemical Examination

We carried out immunohistochemical staining for CD3 and CD20 in the spleen tissues according to Saber, et al. [33]. Slides were first treated with mouse anti-chicken CD3, clone CT-3 (Bio-Rad Lab., Dubai, United Arab Emirates), and CD20 (ThermoFisher Scientific, Waltham, MA, USA) and then assessed as per Amer, et al. [27]. The intensity was expressed by the average grayscale [34].

### 2.12. Statistical Analysis

The data were analyzed using SPSS Version 16 for Windows (SPSS Inc., Chicago, IL, USA). Based on polynomial orthogonal contrasts, one-way ANOVA was applied to calculate linear and quadratic regression equations. The differences between experimental groups were expressed as the mean ± standard deviation (SD) and determined using Duncan’s multiple-range test [35]. The statistical significance of the results was set at (*p <* 0.05).

## 3. Results

### 3.1. Determination of Bioactive Compounds in FO

**Figure 1:**
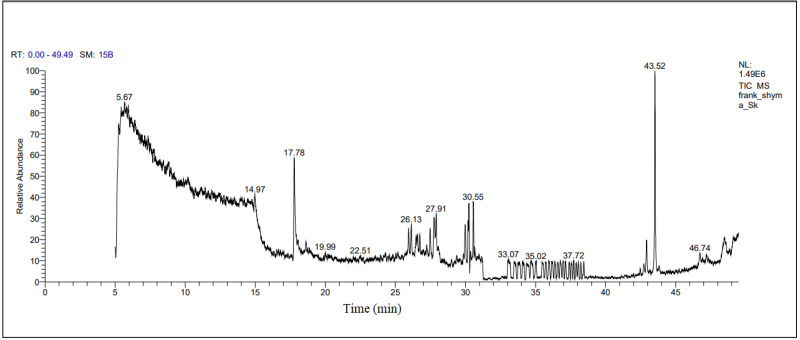
Chromatographic Characteristics of FO Compounds.

Table 2 and Figure 1 list the bioactive compounds in FO identified by GC–MS. The main bioactive compounds include farnesol (12.42%), ç-elemene (12.42%), à-farnesene (12.42%), phenol, bis (1,1-dimethylethyl) (7.15%), phenol, 2,4-bis (1,1-dimethylethyl) 2, 4-di-tert-butylphenol (7.15%), phenol, 3,5-bis (1,1-dimethylethyl) (7.15%), 3-thujanol (3.62%), and 10-undecyn-1-ol (3.50%).

### 3.2. Growth Performance

Table 3 demonstrates the influence of FO supplementation on broilers’ production parameters. In the FO200 group, the BW and BWG were quadratically raised (*p* = 0.04) throughout the starter period. At different FO concentrations, the FCR decreased linearly (*p* = 0.04) and quadratically (*p* = 0.02) with no effect observed on the FI. During the grower period, the BW increased linearly (*p* = 0.001) and quadratically (*p* = 0.02), while the BWG (*p* = 0.001) and FCR (*p* = 0.002) improved linearly at different FO supplementation levels without any effect on the FI (*p* > 0.05) as compared to the FO0 treatment. Different concentrations of FO linearly and quadratically increased the BW and BWG. Compared with the FO0 treatment, FO supplementation decreased FCR (*p* < 0.01) throughout the finisher and overall periods with no effect on the total FI. The final body weight was the highest in the FO200 group and the lowest in the FO0 group (*p* < 0.05).

### 3.3. Percentages of the Dressing, Internal Organs, and Immune Organs

FO supplementation at 200, 400, or 600 mg/kg diet showed no linear or quadratic effect on the percentages of dressing, liver, intestine, gizzard, spleen, and bursa of Fabricus compared to the live weight of birds (*p* > 0.05) (Table 4).

### 3.4. Fatty Acid Composition of Breast Muscle

Different concentrations of FO Supplementation in broilers linearly increased (*p* < 0.01) the percentages of α-linolenic acid (18:3 n-3), eicosapentaenoic acid (20:5 n-3), docosapentaenoic acid (22:5 n-3), docosahexaenoic acid (22:6 n-3), and arachidonic acid, with no effect on the percentage of linoleic acid (18:2 n-6). Compared with the FO0 group, all the FO-supplemented groups showed an increase in the total percentage of n-3 (linear *p* < 0.01, and quadratic *p* = 0.03) and n-6 fatty acids (linear *p* < 0.01, quadratic *p* = 0.01), and also an increased ratio of n-3 to n-6 (linear *p* < 0.01, quadratic *p* = 0.04), with the highest percentage shown at 600 mg/kg diet (Table 5).

### 3.5. Histological Examination

**Figure 2:**
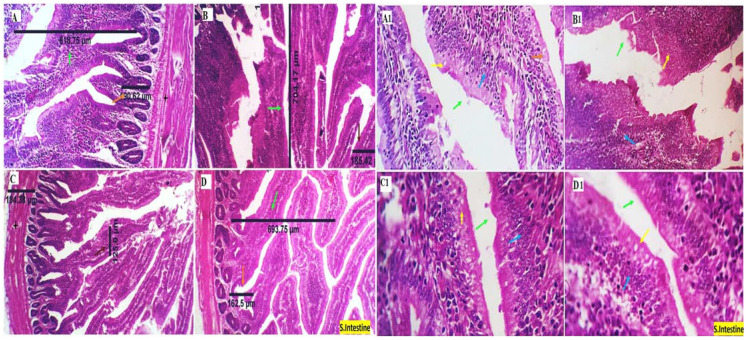
Photomicrograph of the small intestine showing the effect of dietary FO supplementation on different intestinal morphometric measures. VH (green arrows); VW (brown arrows); CD (orange arrows); MCT (black stars). Routine H&E showed a moderate number of goblet cells in FO400 and FO600 groups (16, 17 cells/HPF, respectively) but high numbers in FO0 and FO200 groups (25, 38 cells/HPF, respectively). The FO200 group shows mild villous epithelial stratification with multilayered and proliferated cells having central rounded nuclei and abundant eosinophilic cytoplasm. The normal arrangement of villous cells is seen in other groups. (**A**,**A1**), FO0; (**B**,**B1**), FO200; (**C**,**C1**), FO400; (**D**,**D1**), FO600, H&E × 100, 200, respectively.

Table 6 and Figure 2 illustrate the effects of FO supplementation on the GIT histology of broilers. While FO200 and FO400 groups showed quadratically increased (*p* < 0.01) duodenal villus height (VH), FO400 and FO600 groups showed decreased duodenal villus width (VW) (linear *p* < 0.01 and quadratic *p =* 0.04). In all experimental groups, duodenal crypt depth (CD) was found to be quadratically increased (*p* < 0.01). Compared with the FO0 group, muscular coat thickness (MCT) was increased (*p* < 0.01) in the FO200 and FO400 groups but decreased in the FO600 group. Jejunal VH showed a non-significant increase in the FO200 group and an increase (*p* < 0.01) in the FO400 and FO600 groups compared with the control (FO0). Therefore, dietary FO supplementation linearly and quadratically increased (*p* ≤ 0.01) different jejunum morphometric measures (VW, CD, and MCT). Ileal VH was decreased (*p* < 0.01) in the FO200 and FO600 groups but increased in the FO400 group. FO200, FO400, and FO600 groups showed a linear and quadratic increase in ileal VW and CD. Compared with the control group, ileal MCT increased (*p* ≤ 0.01) in the FO200 group but decreased in the FO400 group. Routine H&E revealed a moderate number of goblet cells in the duodenum of broilers in FO400 and FO600 (16, 17 cells/HPF, respectively) groups but high numbers in FO0 and FO200 (25, 38 cells/HPF, respectively) groups.

### 3.6. Serum Biochemical Parameters

The effect of FO supplementation on the biochemical parameters of broilers is presented in Table 7. Different concentration levels of FO supplementation increased the total protein and growth hormone levels in broilers linearly (*p <* 0.01). Simultaneously, serum albumin showed an increase (*p* < 0.01) in the FO600 group, with insignificant improvement in other treatment groups. Moreover, total globulins increased linearly (*p =* 0.001) in the FO400 and FO600 groups, with insignificant improvement in the FO200 group. The T3 and T4 hormones also increased linearly (*p <* 0.01). These changes were more significant in the FO600 group, followed by the FO400 group. However, glucose and leptin serum levels were not altered (*p* > 0.05).

### 3.7. Immunological Parameters

The influence of FO on the immune status of birds is given in Table 8. Compared with the FO0 group, all FO treatments increased the levels of lysozymes, complement 3, and interleukin 10 linearly and quadratically (*p* ≤ 0.01). The best results were observed at the highest supplementation level (FO600).

### 3.8. Immunohistochemical Examination

**Figure 3:**
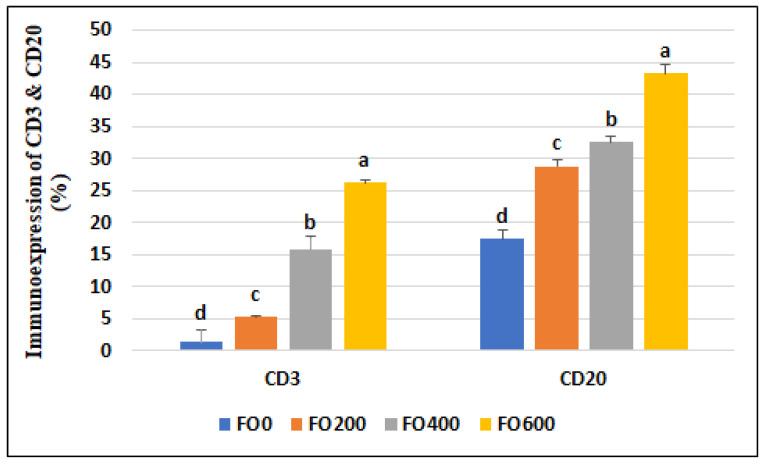
Morphometric analyses of immunostained CD3 and CD20 cells in the spleen of broilers from different experimental groups. Means with different superscripts (^a,b,c,d^) are significant at *p <* 0.05.

**Figure 4:**
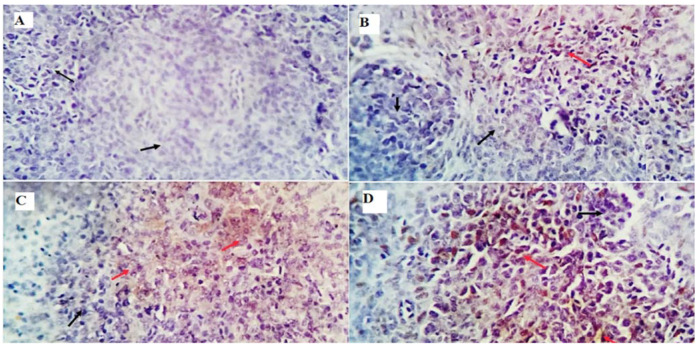
Immunostaining showing positive (red arrows) and negative (black arrows) CD3 cells in the spleen of broilers from different experimental groups. (**A**) FO0; (**B**) FO200; (**C)** FO400; (**D**) FO600.

**Figure 5:**
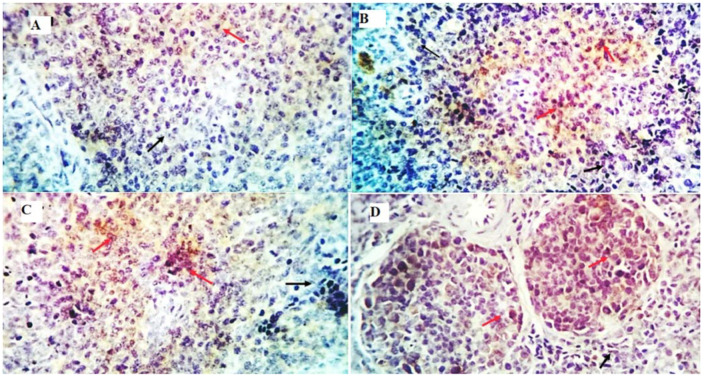
Immunostaining showing positive (red arrows) and negative (black arrows) CD20 cells in the spleen of broilers from different experimental groups. (**A**) FO0; (**B**) FO200; (**C**) FO400; (**D**) FO600.

Morphometric analysis of the spleen sections taken from different experimental groups revealed the following average percentage of positive cells (from three high power fields (HPF)) to CD3 T-cell marker: 1.39, 5.34, 15.9, and 26.3 for FO0, FO200, FO400, and FO600 groups, respectively (Figure 3 and Figure 4). Similarly, the average percentage of positive cells (from three high power fields (HPF)) to CD20 B-cell marker exhibited the following values: 17.4, 28.84, 32.61, and 43.25 for FO0, FO200, FO400, and FO600 groups, respectively (Figure 3 and Figure 5).

## 4. Discussion

The main bioactive compounds identified in FO by GC–MS were farnesol, sesquiterpene constituents, ç-elemene, and α-farnesene along with phenolic compounds, such as bis (1,1-dimethylethyl), phenol,2, 4-bis (1,1-dimethylethyl) 2, 4-di-tert-butylphenol, and phenol, 3, 5-bis (1,1-dimethylethyl). Our results revealed a positive effect of dietary FO supplementation on BWG and FCR of broiler chickens without impacting their feed intake. The most significant results were observed in the group supplemented with 200 mg/kg FO, followed by the group supplemented with 400 mg/kg FO. These inclusive improvements in the growth parameters may be attributed to the following various reasons: (1) the overall good health of birds and improved gastrointestinal tract morphology suggested by an increase in villus height, crypt depth, and absorptive surface area [11,36]; (2) improved absorption of essential nutrients (calcium, phosphorus, and iron) [37]; (3) stimulated activities of gastrointestinal tract enzymes, reduced gas flow, and enhanced gastric juice secretion and flow, leading to increased nutrient digestibility of dry matter and organic matter [38]. Furthermore, the improved growth reported in this study could be due to the increased secretion of growth and thyroid hormones along with improved intestinal histomorphology.

Improved feed efficiency and performance of broilers supplemented with *Boswellia serrata* (BS) were the results of the better configuration of intestinal villi, gastric microflora, and the overall health of broilers [11,39]. Moreover, BS supplementation in broiler diets at 0.5, 1, and 1.5 g/kg increased their body weight and weight gain linearly; therefore, improving their FCR linearly and quadratically [40]. Previous studies have shown that enriching rabbit diets with 0.25, 0.50, 0.750, and 1.00 g/kg BS improved their BWG and FCR [41]. However, this was in contrast with other researchers who observed a non-significant effect of olibanum (*Boswellia thurifera*) supplementation at 0.01, 0.015, 0.02, 0.03, or 0.05% and BS resin supplementation at 1.5, 2, or 2.5% on the performance parameters of broilers [36,42]. Moreover, Tabatabaei, et al. [43], reported that during the grower period, broilers supplemented with 0.5% BS exhibited the lowest FCR compared with the control birds.

Our results revealed that supplementation of diets with 200, 400, or 600 mg/kg FO showed no linear or quadratic effect on the percentages of dressing, internal organs, and immune organs’ weight. These findings conferred with the results of Ismail, et al. [41], who confirmed that most traits relating to the composition of rabbit carcass and edible organs were insignificantly affected by diets supplemented with BS. However, our results disagree with that of Mohamed, et al. [40], who reported that supplementing different BS levels to broiler diets improved relative weights of the liver, heart, spleen, bursa, and thymus gland while quadratically increasing the relative weights of gizzard and giblet compared with the control. Moreover, Al-Yasiry, et al. [42] reported good carcass quality of chickens fed with 2.0–2.5% BS-containing diets compared with that of non-treated chickens. The difference between our results and the previous results may be attributed to the form of the additive. While we used BS oil, the previous studies used the whole plant, which definitely differed in composition.

Enriching broiler diets with PUFA leads to improved meat quality [44]. Various research studies have been conducted to change the fatty acid content in poultry meat [45,46]. Enriching broiler diets with herbal extracts and oils has received much attention due to their application in enhancing production parameters and poultry health [47]. In this work, dietary FO supplementation enriched the breast muscle with n-3 PUFA, mainly the α-linolenic acid, eicosapentaenoic acid, docosapentaenoic acid, and docosahexaenoic acid, and also n-6 PUFA, particularly the arachidonic acid. It also increased the n-3/n-6 ratio, favoring the acceptance of consumers. The positive effect of FO supplementation on the fatty acid composition of breast muscles may be attributed to different boswellic acids, terpenoids, polyphenols and flavonoids of BS. These can improve the composition of fatty acids, and thereby, the quality of meat [48]. The fatty acid content in broilers’ meat is affected by their diet composition [49] and genetic structure [50]. Our results were in line with the results of previous research in broilers, which demonstrated the positive effect of 1.5, 2, or 2.5% BS resin supplementation on the percentage of PUFA, the sum of total fatty acids, n-3/n-6 saturation, hypocholesterolemic/hypercholesterolemic ratio in breast, abdominal fat, and drumstick muscles in broilers [48]. Furthermore, Nkukwana, et al. [51] showed that diets supplemented with *Moringa oleifera* leaves increased fatty acids in poultry meat, while broilers fed with *Lippia javanica*, consisting of the highest terpenes, showed increased levels of oleic acid content in their drumstick muscle [52].

The small intestine in poultry animals is an important gut organ required for nutrient digestion and absorption. In this work, supplementing broilers’ diets with different concentrations of FO increased different morphometric measures of the duodenum and jejunum (VH, VW, CD, and MCT), ileal VW, CD, and MCT. The ileal VH was reduced in the FO200 and FO600 groups but increased in the FO400 group. Lower VH with a linear increase in the VW of the ileum indicated that with the increase in the FO levels, the absorptive surface area also increased, which has the advantage of more nutrient absorption. Moreover, polyphenols and some terpenes decrease extreme oxidative stress by reducing plasma lipid peroxidation [53]. These bioactivities enhance gut health, thus improving the overall health of the animal. Several aromatic plant biostimulants have been reported to enhance intestinal morphology and expression of tight junction proteins, benefiting animals [54,55,56]. BS resin supplementation in broiler diets at 2 or 2.5% levels decreased crypt depth and increased the ratio of villus: crypt without altering VH, while BS levels at 3 and 4% increased duodenal length [54]. Tabatabaei [36], reported that adding different increments of olibanum at 0.01, 0.015, 0.02, 0.03, and 0.05 increased VH and crypt depth of duodenum and jejunum insignificantly but increased ileum VH significantly. The epithelium turnover was positively affected by the increase in intestinal villi height and villus crypt ratio. The reduction in intestinal crypts indicated a decrease in the exchange of enterocytes and also a lower requirement for tissue development [55,56].

Although the experimental data showed no effect of FO supplementation on serum levels of glucose and leptin, an increase was observed in the serum levels of albumin, total globulins, total proteins, thyroxin, and growth hormones. T3 and T4 are produced by the thyroid gland and are essential for regulating many metabolic and feeding processes. They also control the gain rate through several metabolic mechanisms [57]. Higher serum levels of T3 and T4 hormones may result in greater BWG [58]. The anterior pituitary gland’s somatotroph cells secrete growth hormone (GH), which is essential for numerous biological activities. It manages animal growth and the progression of tissue levels [59,60]. The results of growth and thyroid hormones in our study explain the improved growth by FO. A higher globulin level is a valuable index for higher immune response and antibody production [39,61]. *Boswellia serrata* encourages the function of the thyroid gland, leading to the upregulation of metabolism and an increase in the basal metabolic rate. Our results are consistent with previous results, where the supplementation of BS at 0.5, 1, and 1.5 g/kg increased the globulin level in broilers [40,42]. Additionally, the enrichment of drinking water with different concentrations of Frankincense powder at 0.25, 0.50, 0.75, and 1 g/Liter significantly increased the plasma concentration of total proteins [62]. In contrast to our work, the supplementation of *Boswellia serrata* resin (BSR) in broiler diets at 1.5, 2, or 2.5% did not alter the values of total serum levels of protein, albumin, and globulins [39]. Another study confirmed that rabbits fed with a BS-enriched diet showed lower albumin levels and A/G ratio compared with the control diet [41].

Our investigation showed an increase in the activities of lysozyme, complement 3, and interleukin 10, with the highest increase observed in the uppermost supplementation level (FO600). Due to its effectiveness and well-established role in the immune process, lysozyme is considered an essential component of non-specific humoral immunity; it has a bactericidal impact and can activate the complement system and phagocytic activity, leading to the destruction of the glycosidic bonds of *E.coli* and *Staphylococcus* walls, preventing infection and disease [63]. Lymphocyte subpopulations are identified by specific cell surface biomarkers. After antigen recognition, the T-cell co-receptor CD3 initiates a signaling cascade that activates helper and cytotoxic T cells [64]. A B-lymphocyte surface antigen, CD20, regulates B-cell activity, differentiation, and proliferation [65]. The present study showed that FO supplementation in broiler diets led to significant upregulation of the immunoexpression of CD3 and CD20 genes in the spleen tissue. These results indicate the immunomodulatory effects of FO supplementation in broiler chickens. FO supplementation results in the activation of B- and T-lymphocytes and the production of IgG and IgM antibodies that protect the body from bacterial and viral infection [5,66]. Mikhaeil, et al. [12] performed a lymphocyte proliferation assay and reported an intense immunostimulant activity of FO, which revealed 90% lymphocyte transformation.

Undoubtedly, the immunomodulatory effect of FO is due to its bioactive compound profile. Farnesol, a bioactive compound in FO, has exhibited antibiofilm, fungicidal, antitumor, and anticancer properties [67,68]. Sachivkina, et al. [69] showed that farnesol increased the resistance against yeast-like fungi, suggesting its ability as an antimicrobial compound [70,71,72,73]. Moreover, farnesol stimulates the NF-kB pathway via MEK1/2-ERK1/2-MSK1-dependent phosphorylation of p65, consequently stimulating cytokine production, including IL-6 and IL-1α [74]. Additionally, sesquiterpene constituents, such as c¸-elemene and α-farnesene, exhibit immunomodulatory effects by modulating the anti-inflammatory response through the inhibition of prostaglandin, lipoxygenase, and leukotriene biosynthesis [75]. It is well known that terpenes inhibit bacterial cell division [76] and are quorum sensing (QS) inhibitors. QS is an intracellular bacterial communication system that permits various activities, for example, biofilm formation and expression of virulence factors [77]. Phenol-2, 4-bis (1,1-dimethylethyl) is used as an antioxidant, UV stabilizer, or light protection agent [78,79]. Besides its antimicrobial activity [79,80,81,82], Ren, et al. [78] confirmed that phenol-2, 4-bis (1,1-dimethylethyl) from *Pseudomonas fluorescens* TL-1 possessed antifungal activity. In ethanol-induced gastric damage models, phenol, 3, 5-bis (1,1-dimethylethyl) and phenol-2, 4-bis (1,1-dimethylethyl) were established to exhibit anti-inflammatory and gastroprotective activities [76].

## 5. Conclusions

Our results have suggested that supplementing broiler diets with up to 600 mg kg^−1^ of FO enhances growth performance by stimulating the secretion of growth and thyroid hormones and improving intestinal histomorphology. Dietary FO enhances the birds’ immunity by increasing serum levels of lysozyme, interleukin 10, and complement 3 and also by increasing the expression of the immunolabelling indexes of CD3 and CD20. Dietary FO enriched the breast muscles of birds with n-3 and n-6 PUFA. Thus, FO can be used as a native growth promoter and immune-stimulating agent for broilers. In light of serious issues such as antimicrobial resistance in global animal production, the use of functional feed additives is of paramount interest. Our study on broilers validates the prophylactic benefits of FO in the livestock industry.

## Figures and Tables

**Table 1:** Proximate Chemical Composition of the Experimental Diets (%).

Ingredients	Starter Period (4–10 d)	Grower Period (11–23 d)	Finisher Period (24–35 d)
Yellow corn	55.9	59.3	62
Corn gluten, 60%	3.935	5.275	6.07
Soybean meal, 48%	33.42	28.1	23.825
Soybean oil	2.2	3	4
Calcium dibasic phosphate	1.5	1.4	1.3
Calcium carbonate	1.2	1.2	1.1
Common salt	0.15	0.15	0.15
DL-methionine, 98%	0.4	0.3	0.33
Premix *	0.3	0.3	0.3
Lysine HCl, 78%	0.47	0.45	0.40
Phytase	0.005	0.005	0.005
Threonine	0.1	0.1	0.1
Choline	0.07	0.07	0.07
Antimycotoxin	0.1	0.1	0.1
Na_2_CO_3_	0.25	0.25	0.25
Chemical composition			
Crude protein%	23.05	21.52	20.15
ME (Kcal/kg)	3004	3101	3201
Calcium %	0.941	0.904	0.832
Available phosphorus %	0.481	0.448	0.417
Lysine %	1.46	1.31	1.16
Methionine %	0.721	0.610	0.626
Threonine %	0.824	0.765	0.713

* Premix per kg of diet: Vit. A, 1500 IU; Vit. E, 10 mg; Vit. D3, 200 IU; Vit. K3, 0.5 mg; thiamine, 1.8 mg; pantothenic acid, 10 mg; riboflavin, 3.6 mg; pyridoxine, 3.5 mg; niacin, 35 mg; folic acid, 0.55 mg; biotin, 0.15 mg; cobalamin, 0.01 mg; Cu, 8 mg; Fe, 80 mg; Mn, 60 mg; Zn, 40 mg; Se, 0.15 mg; I, 0.35 mg.

**Table 2:** Gas Chromatography–Mass Spectrometry (GC–MS) Analysis of FO.

Bioactive Compounds	Retention Time	Peak Area %
Farnesol	43.53	12.42
ç-Elemene	43.53	12.42
à-Farnesene	43.53	12.42
Phenol, bis(1,1-dimethylethyl)	17.78	7.15
Phenol, 2,4-bis(1,1-dimethylethyl) 2,4-di-tert-butylphenol	17.78	7.15
Phenol, 3,5-bis(1,1-dimethylethyl)	17.78	7.15
3-Thujanol	30.25	3.62
Bicyclo [3.1.0] hexan-3-ol, 4-methyl-1-(1-methylethyl)	30.25	3.62
Methyl 11,12-tetradecadienoate	30.25	3.62
10-Undecyn-1-ol	30.56	3.50
(3-Cyclopropylbicyclo [4.1.0] hept-7-Yl)methanol	30.56	3.50
E,E-6,11-Tridecadien-1-ol acetate	30.56	3.50
Bicyclo [4.1.0]heptane,-3-cyclopropyl,-7-hydroxymethyl, trans	30.56	3.50
Dichloroacetic acid, dodec-9-ynyl	30.56	3.50

**Table 3:** Effects of Dietary FO Supplementation on Growth Performance of Broiler Chickens During the Feeding Periods.

Trait Measured	FO0	FO200	FO400	FO600	Regression	
					Linear	Quadratic
Initial BW (g)	99.44 ± 0.01	99.38 ± 0.63	98.75 ± 0.01	99.17 ± 0.72	0.271	0.404
Starter period (4–10 days)						
BW (g)	303.25 ± 24.11 ^b^	356.53 ± 14.02 ^a^	329.83 ± 15.13 ^ab^	335.28 ± 10.01 ^ab^	0.145	0.04
BWG (g)	203.81 ± 24.11 ^b^	257.15 ± 14.29 ^a^	231.08 ± 15.13 ^ab^	236.11 ± 10.66 ^ab^	0.141	0.04
FI (g)	271.85 ± 5.59	273.12 ± 7.60	258.96 ± 11.39	267.08 ± 4.16	0.189	0.462
FCR	1.34 ± 0.13 ^a^	1.06 ± 0.03 ^b^	1.13 ± 0.11 ^b^	1.13 ± 0.05 ^b^	0.039	0.024
Grower period (11–23 days)						
BW (g)	1073.33 ± 50.07 ^b^	1272.00 ± 12.53 ^a^	1228.61 ± 55.09 ^a^	1289.06 ± 37.52 ^a^	0.001	0.022
BWG (g)	770.08 ± 29.98 ^b^	915.47 ± 23.17 ^a^	898.78 ± 63.99 ^a^	953.78 ± 27.99 ^a^	0.001	0.08
FI (g)	1205.19 ± 29.66	1216.04 ± 100.87	1125.21 ± 38.56	1175.83 ± 29.13	0.265	0.568
FCR	1.57 ± 0.03 ^a^	1.33 ± 0.15 ^b^	1.26 ± 0.12 ^b^	1.23 ± 0.01 ^b^	0.002	0.09
Finisher period (24–35 days)						
BW (g)	1762.22 ± 56.13 ^b^	2209.72 ± 8.67 ^a^	2186.11 ± 59.12 ^a^	2161.67 ± 88.93 ^a^	<0.01	<0.01
BWG (g)	688.89 ± 13.84 ^c^	937.72 ± 21.09 ^ab^	957.50 ± 41.89 ^a^	872.61 ± 55.19 ^b^	<0.01	<0.01
FI (g)	1487.78 ± 125.59	1607.08 ± 134.10	1452.92 ± 53.16	1455.00 ± 66.14	0.362	0.345
FCR	2.16 ± 0.16 ^a^	1.71 ± 0.11 ^b^	1.52 ± 0.04 ^b^	1.67 ± 0.16 ^b^	<0.01	<0.01
Overall performance						
Final BW, g	1762.22 ± 56.13 ^b^	2209.72 ± 8.67 ^a^	2186.11 ± 59.12 ^a^	2161.67 ± 88.93 ^a^	<0.01	<0.01
Total BWG, g	1662.78 ± 56.13 ^b^	2110.35 ± 8.23 ^a^	2087.36 ± 59.12 ^a^	2062.50 ± 89.64 ^a^	<0.01	<0.01
Total FI, g	2964.81 ± 159.15	3096.25 ± 238.20	2837.08 ± 78.07	2897.92 ± 58.05	0.273	0.697
FCR	1.78 ± 0.04 ^a^	1.47 ± 0.11 ^b^	1.36 ± 0.03 ^b^	1.41 ± 0.07 ^b^	<0.01	<0.01

^a,b,c^ Means within the same row carrying different superscripts were significantly different at *p* < 0.05.

**Table 4:** Effect of FO Supplementation on the Percentages of Dressing, Internal Organs, and Immune Organs (%).

	FO0	FO200	FO400	FO600	Regression	
					Linear	Quadratic
Dressing %	58.577 ± 0.53	57.573 ± 0.90	57.70 ± 0.35	58.13 ± 0.94	0.367	0.220
Liver %	2.66 ± 0.40	2.47 ± 0.34	2.38 ± 0.48	2.44 ± 0.44	0.512	0.620
Spleen %	0.13 ± 0.05	0.10 ± 0.02	0.12 ± 0.02	0.15 ± 0.08	0.635	0.302
Intestine %	5.43 ± 0.35	6.06 ± 0.43	5.91 ± 0.58	5.21 ± 0.42	0.518	0.05
Gizzard %	1.78 ± 0.31	2.24 ± 0.28	1.92 ± 0.08	1.73 ± 0.20	0.483	0.05
Bursa %	0.13 ± 0.02	0.15 ± 0.04	0.16 ± 0.03	0.11 ± 0.01	0.611	0.084

**Table 5:** Effect of FO Supplementation on the Fatty Acid Composition of Breast Muscle.

	FO0	FO200	FO400	FO600	Regression	
					Linear	Quadratic
ALA %	0.033 ±0.01 ^b^	0.053 ±0.01 ^a^	0.056 ±0.01 ^a^	0.066 ±0.01 ^a^	0.001	0.290
EPA %	0.026 ±0.01 ^c^	0.040 ±0.00 ^b^	0.046 ±0.01 ^b^	0.050 ±0.01 ^a^	<0.01	0.067
DPA %	0.023 ±0.01 ^b^	0.040 ±0.01 ^a^	0.040 ±0.01 ^a^	0.046 ±0.01 ^a^	<0.01	0.067
DHA %	0.016 ±0.01 ^b^	0.030 ±0.01 ^a^	0.036 ±0.01 ^a^	0.036 ±0.01 ^a^	0.001	0.050
LA %	0.826 ±0.06	0.890 ±0.10	0.863 ±0.01	0.860 ±0.01	0.656	0.374
AA %	1.14 ±0.04 ^b^	1.20 ±0.08 ^b^	1.28 ±0.01 ^a^	1.29 ±0.02 ^a^	0.002	0.393
n-3 (%).	0.096 ±0.02 ^b^	0.163 ±0.01^a^	0.176 ±0.02^a^	0.193 ±0.02 ^a^	<0.01	0.03
n-6 (%).	1.97 ±0.05 ^c^	2.09 ±0.03 ^b^	2.15 ±0.01 ^a^	2.15 ±0.02 ^a^	<0.01	0.01
n-3: n-6 ratio	0.049 ±0.01 ^b^	0.078 ±0.002 ^a^	0.082 ±0.01 ^a^	0.089 ±0.01 ^a^	<0.01	0.04

^a,b,c^ Mean values in the same row with different superscripts differ significantly (*p* < 0.05). ALA: alphalinolenic acid (18:3 n-3), EPA: eicosapentaenoic acid (20:5 n-3), DPA: docosapentaenoic acid (22:5 n-3), DHA: docosahexaenoic acid (22:6 n-3), LA: linoleic acid (18:2 n-6), AA: arachidonic acid (20:4 n-6), n-3 (% of total fatty acids): omega-3 PUFA, n-6 (% of total fatty acids): omega-6 PUFA.

**Table 6:** Effect of Dietary FO Supplementation on Intestinal Morphometry.

	FO0	FO200	FO400	FO600	Regression	
					Linear	Quadratic
Duodenum						
VH µm	690.68 ± 13.0 ^c^	814.78 ± 3.94 ^a^	765.07 ± 22.55 ^b^	663.11 ± 36.42 ^c^	0.05	<0.01
VW µm	181.57 ± 3.73 ^a^	184.47 ± 4.00 ^a^	121.82 ± 3.05 ^c^	134.42 ± 3.08 ^b^	<0.01	0.043
CD µm	140.56 ± 3.24 ^c^	183.42 ± 6.60 ^a^	150.04 ± 6.39 ^bc^	159.67 ± 3.45 ^b^	0.110	<0.01
MCT µm	140.75 ± 2.10 ^c^	254.81 ± 10.90 ^a^	179.56 ± 5.404 ^b^	125.03 ± 2.88 ^d^	<0.01	<0.01
Jejunum						
VH µm	811.11 ± 23.5 ^c^	862.11 ± 16.3 ^bc^	897.38 ± 45.85 ^b^	995.43 ± 12.77 ^a^	<0.01	0.180
VW µm	115.36 ± 8.48 ^d^	266.06 ± 10.62 ^a^	158.32 ± 7.06 ^c^	247.52 ± 5.66 ^b^	<0.01	<0.01
CD µm	112.76 ± 3.06 ^c^	190.68 ± 14.98 ^a^	154.90 ± 7.56 ^b^	160.45 ± 4.78 ^b^	0.002	<0.01
MCT µm	115.15 ± 3.42 ^b^	166.06 ± 6.19 ^a^	116.28 ± 2.50 ^a^	118.98 ± 2.81 ^a^	0.006	<0.01
Ileum						
VH µm	676.29 ± 30.4 ^b^	522.81 ± 8.32 ^c^	765.23 ± 12.76 ^a^	474.49 ± 14.72 ^d^	<0.01	<0.01
VW µm	111.73 ± 3.72 ^c^	126.89 ± 10.06 ^b^	210.60 ± 7.63 ^a^	205.00 ± 6.98 ^a^	<0.01	0.04
CD µm	106.89 ± 2.92 ^c^	165.94 ± 7.50 ^a^	134.92 ± 5.83 ^b^	137.26 ± 7.80 ^b^	0.006	<0.01
MCT µm	147.36 ± 2.63 ^b^	301.06 ± 29.31 ^a^	112.81 ± 2.63 ^c^	139.43 ± 4.54 ^bc^	0.001	<0.01

^a,b,c,d^ Mean values in the same row with different superscripts differ significantly (*p <* 0.05).

**Table 7:** Effect of Dietary FO Supplementation on Serum Biochemical Parameters.

	FO0	FO200	FO400	FO600	Regression	
					Linear	Quadratic
Total proteins (g/dL)	3.393 ± 0.27 ^d^	3.983 ± 0.06 ^c^	4.453 ± 0.36 ^b^	5.240 ± 0.13 ^a^	<0.01	0.497
Albumin (g/dL)	1.250 ± 0.03 ^b^	1.347 ± 0.01 ^b^	1.730 ± 0.39 ^ab^	1.943 ± 0.44 ^a^	0.012	0.739
Total globulins (g/dL)	2.143 ± 0.026 ^c^	2.637 ± 0.06 ^bc^	2.723 ± 0.06 ^b^	3.297 ± 0.51 ^a^	0.001	0.816
GH (ng/mL)	2.73 ± 0.35 ^c^	4.03 ± 0.47 ^b^	5.00 ± 0.36 ^a^	5.23 ± 0.40 ^a^	<0.01	0.050
T3 (ng/mL)	3.39 ± 0.17 ^c^	4.26 ± 0.06 ^b^	4.69 ± 0.36 ^ab^	5.10 ± 0.35 ^a^	<0.01	0.176
T4 (ng/mL)	19.41 ± 0.21 ^d^	21.65 ± 0.91 ^c^	23.31 ± 0.89 ^b^	25.35 ± 0.79 ^a^	<0.01	0.822
Glucose (mg/dL)	336.67 ± 2.08	340.33 ± 5.13	340.00 ± 3.61	341.33 ± 3.06	0.184	0.594
Leptin (ng/mL)	2.10 ± 0.06	1.92 ± 0.24	1.94 ± 0.19	2.20 ± 0.04	0.440	0.050

^a,b,c,d^ Mean values in the same row with different superscripts differ significantly (*p <* 0.05).

**Table 8:** Effect of Dietary FO Supplementation on the Immune Status of Broiler Chickens.

	FO0	FO200	FO400	FO600	Regression	
					Linear	Quadratic
Lysozyme enzyme (µg/mL)	133.00 ±2.65 ^c^	172.67 ±14.43 ^b^	184.00 ±8.00 ^ab^	192.00 ±1.00 ^a^	<0.01	0.011
Complement 3 (g/L)	1.063 ±0.02 ^d^	1.190 ±0.02 ^c^	1.257 ±0.02 ^b^	1.293 ±0.02 ^a^	<0.01	0.003
IL10 (ug/mL)	1.733 ±0.51 ^c^	3.500 ±0.35 ^b^	3.967 ±0.21 ^ab^	4.400 ±0.26 ^a^	<0.01	0.011

^a,b,c,d^ Mean values in the same row with different superscripts differ significantly (*p* < 0.05).

## Data Availability

The datasets generated or analyzed during the current study are not publicly available but are available from the corresponding author upon reasonable request.
